# Evaluation of four sampling devices for *Burkholderia pseudomallei* laboratory aerosol studies

**DOI:** 10.1371/journal.pntd.0009001

**Published:** 2021-02-01

**Authors:** Michael Schuit, Sierra Gardner, Jill Taylor, Paul Dabisch

**Affiliations:** 1 National Biodefense Analysis and Countermeasures Center, Operated by BNBI for the U.S. Department of Homeland Security Science and Technology Directorate, Frederick, Maryland, United States of America; 2 School of Systems Biology, George Mason University, Manassas, Virginia, United States of America; Mahidol University, THAILAND

## Abstract

Previous field and laboratory studies investigating airborne *Burkholderia pseudomallei* have used a variety of different aerosol samplers to detect and quantify concentrations of the bacteria in aerosols. However, the performance of aerosol samplers can vary in their ability to preserve the viability of collected microorganisms, depending on the resistance of the organisms to impaction, desiccation, or other stresses associated with the sampling process. Consequently, sampler selection is critical to maximizing the probability of detecting viable microorganisms in collected air samples in field studies and for accurate determination of aerosol concentrations in laboratory studies. To inform such decisions, the present study assessed the performance of four laboratory aerosol samplers, specifically the all-glass impinger (AGI), gelatin filter, midget impinger, and Mercer cascade impactor, for collecting aerosols containing *B*. *pseudomallei* generated from suspensions in two types of culture media. The results suggest that the relative performance of the sampling devices is dependent on the suspension medium utilized for aerosolization. Performance across the four samplers was similar for aerosols generated from suspensions supplemented with 4% glycerol. However, for aerosols generated from suspensions without glycerol, use of the filter sampler or an impactor resulted in significantly lower estimates of the viable aerosol concentration than those obtained with either the AGI or midget impinger. These results demonstrate that sampler selection has the potential to affect estimation of doses in inhalational animal models of melioidosis, as well as the likelihood of detection of viable *B*. *pseudomallei* in the environment, and will be useful to inform design of future laboratory and field studies.

## Author summary

*Burkholderia pseudomallei* is a type of bacteria that can cause a severe disease called melioidosis in humans, and it is known to be transmitted by aerosols in some cases. In this study, we compare the performance of four aerosol samplers for *B*. *pseudomallei* aerosolized from different types of liquid suspensions. We found that differences in sampler performance were dependent on the composition of the suspension from which the bacteria was aerosolized, with samplers that collect onto dry substrates (25mm gelatin filters and a Mercer cascade impactor) performing poorly when the initial suspension contained nothing to protect the bacteria against desiccation. The results of this study will help inform comparisons between previous studies of *B*. *pseudomallei* in aerosols that used different methods, and will be useful to inform sampler choices for future studies, both in laboratory settings with controlled conditions and field studies with aerosols of unknown composition.

## Introduction

The gram-negative bacterium *B*. *pseudomallei*, the causative agent of the disease melioidosis, is a soil saprophyte endemic to Southeast Asia, northern Australia, and other tropical regions around the world [1]. Melioidosis is a significant public health concern, with estimates of upwards of 100,000 cases occurring worldwide annually [2–5]. Direct contact with contaminated soil or water is considered to be the primary route of exposure, but there is evidence that aerosol transmission also contributes to the spread of melioidosis. While one air sampling study in the endemic region found no evidence of *B*. *pseudomallei* in air samples[6], culturable *B*. *pseudomallei* has been isolated from outdoor air samples collected downwind from known environmental reservoirs and upwind of cases of melioidosis with respiratory symptoms [7,8], and *B*. *pseudomallei* genetic material has been found in air samples during a typhoon season in Taiwan [9]. Furthermore, airborne transmission has been demonstrated for other *Burkholderia* species, including *B*. *cepacia* and *B*. *cenocepacia*, [10–13].

Studies to assess the hazard posed by an airborne pathogen rely on aerosol sampling devices to collect material for subsequent analyses. However, previous studies have demonstrated that aerosol samplers differ in their ability to collect and preserve the viability of collected microorganisms [14–17], complicating comparisons of results from studies that have utilized different samplers. Differences in sampler performance have the potential to bias study outcomes, including conclusions about the presence or absence of the microorganism in naturally occurring aerosols in a field study or assessments of the inhalational virulence in animal models of disease. Previous studies of aerosols containing *Burkholderia* spp. have employed a wide variety of sampling devices to collect material for analyses. Studies have detected *Burkholderia* spp. in field studies using both high flow air samplers [8,18–20] as well as lower flow devices, specifically impingers and filters [7,9]. Laboratory studies examining the effects of inhaled *B*. *pseudomallei* in animal models of disease have predominantly utilized liquid impingers to quantify the concentration of the bacterial aerosols and estimate the dose received [21–33].

Despite the public health concerns surrounding *B*. *pseudomallei* and its potential for aerosol transmission, as well as the potential for sampler selection to affect study outcomes, only one study was identified that reported data comparing the performance of aerosol samplers with this microorganism [22]. That study examined common laboratory aerosol samplers and demonstrated that the Mercer cascade impactor, gelatin filter, and AGI performed equivalently for quantifying concentrations of aerosolized *B*. *pseudomallei* [22]. The aim of the present study was to extend the findings of this previous study by assessing the performance of additional aerosol samplers commonly utilized in the laboratory for sampling aerosolized *B*. *pseudomallei*, as well as to assess the effect of the medium in which the microorganism is suspended prior to aerosolization on sampler performance. Data on the relative performance of aerosol sampling devices produced by this study may be useful to inform sampling strategies for future laboratory studies examining the inhalational hazard posed by *B*. *pseudomallei*, as well as selection of sampling devices for field studies seeking to quantify concentrations of naturally occurring outdoor aerosols containing viable *B*. *pseudomallei*.

## Methods

### Bacteria

*B*. *pseudomallei* 1026b was obtained from Battelle Memorial Institute (BMI) in Columbus, OH. BMI had obtained the stock directly from the Mahidol Oxford Tropical Medicine Research Unit in Bangkok, Thailand. *B*. *pseudomallei* 1026b was originally isolated in 1993, and has since become a commonly used isolate for inhalation studies using animal models of melioidosis [26,32–36]. The day prior to an experiment, 200 μL of thawed single use frozen aliquots of *B*. *pseudomallei* 1026b were used to inoculate 50 mL of LB-Lennox (LB) Broth (Teknova) in a 200 mL baffled flask. Cultures were incubated with shaking at 225 rpm for 18 h at 37°C to produce stationary phase bacteria. The suspension containing stationary phase bacteria was diluted 1:10 in LB broth or LB broth supplemented with 4% glycerol (LB4G) and stored at room temperature for up to two hours until use. Both LB broth and LB4G, or media with similar compositions, are commonly used for culturing *B*. *pseudomallei* for aerosol experiments [27,37–41]. In the present study, aerosol starting material suspensions in LB had a mean titer of 8.69 ± 0.08 Log colony forming units (CFU)/mL, and suspensions in LB4G had a mean titer of 8.71 ± 0.08 Log CFU/mL. All work with *B*. *pseudomallei* was conducted in a Biosafety Level 3 (BSL-3) laboratory.

Colony counts were used to estimate concentrations of viable *B*. *pseudomallei* in aerosol starting materials and in aerosol samples. Samples were diluted in phosphate buffered saline (PBS; Gibco) containing 0.002% Tween-80 (Millipore Sigma) (PBST), plated in triplicate on sheep blood agar (SBA) plates, inverted, and incubated for 44–48 h at 37°C before counting colonies.

### Tracer

Tests to assess physical performance of the aerosol sampling devices utilized a non-labile tracer, specifically 1 μm FluoroMax green polystyrene latex (PSL) microspheres (PN G0100, ThermoFisher Scientific), suspended in LB or LB4G. Total fluorescence in samples was measured using a Glomax Multi Jr fluorescence reader (Promega) with excitation and emission wavelengths of 460 nm and 515–570 nm, respectively. Results are reported as relative fluorescence units (RFU).

### Aerosol test system and samplers

**Fig 1:**
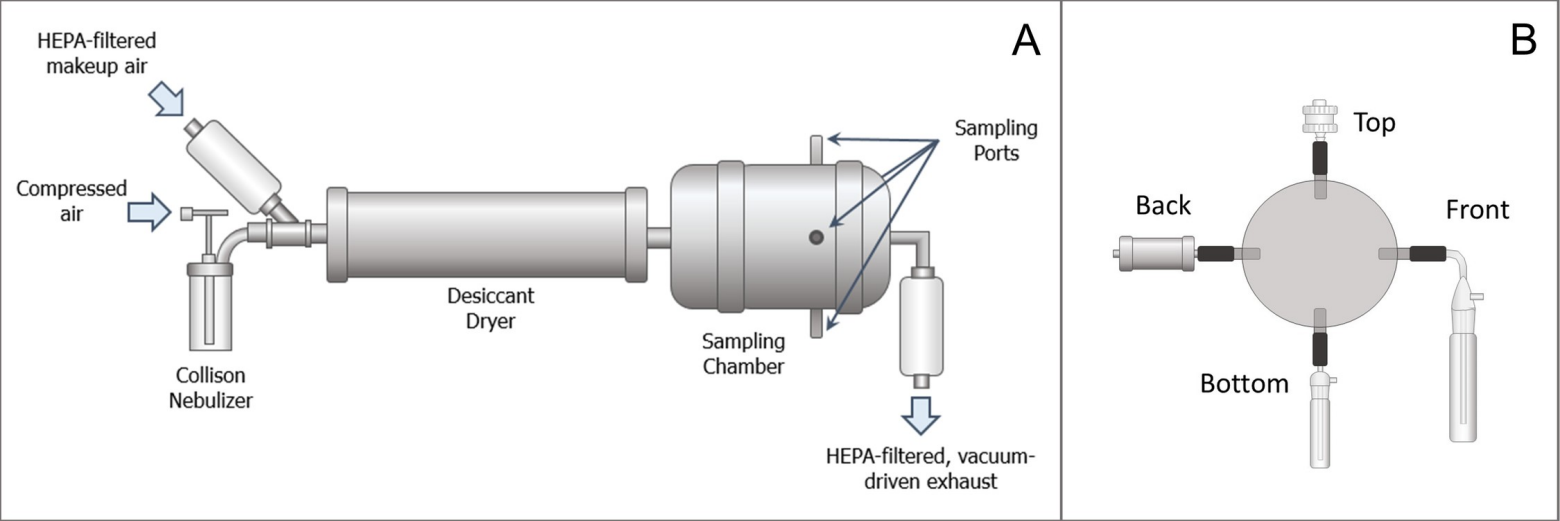
Aerosol test system. (A) Side view: a Collison nebulizer generated aerosols that passed through a desiccant dryer before entering the sampling chamber. The aerosol was sampled from four ports spaced evenly around the circumference of the sampling chamber; (B) Cross-sectional view at the plane of sampling ports: Samplers were connected to the sampling chamber as shown to prevent the need for any additional bends in the sampling inlets. Clockwise from top: gelatin filter in delrin holder, AGI, midget impinger, and Mercer cascade impactor.

A test system consisting of an aerosol generator, a desiccant dryer, and chamber with multiple sampling ports was constructed and used to compare the performance of four devices for sampling *B*. *pseudomallei* aerosols (Fig 1). Aerosols were generated into the system using a 3-jet Collison nebulizer (CH Technologies) controlled with a mass flow controller (Alicat Scientific) at an average flow of 8.4 ± 0.1 Lpm. A desiccant drier (In-Tox Products) was situated downstream of the aerosol generator to aid in droplet evaporation and equilibration before the aerosol entered the sampling chamber. The sampling chamber was constructed of 10.2 cm diameter stainless steel sanitary pipe with four 1.3 cm sampling ports evenly spaced around the circumference of the sampling chamber. Aerosol sampling devices were connected to the ports on the sampling chamber with conductive tubing. Perforated stainless steel plates located upstream and downstream of the sampling ports facilitated uniform distribution of the airflow and aerosol within the sampling chamber. The total exhaust airflow of the system consisted of the flows of the individual samplers and additional HEPA-filtered vacuum pump-driven exhaust, and was maintained at an average of 19.9 ± 0.2 Lpm. A HEPA-filter (TSI Inc.) located immediately upstream of the desiccant dryer allowed for passive entry of additional dilution air required to balance the supply and exhaust airflows.

The performance of four types of laboratory aerosol samplers were evaluated in this study: All Glass Impingers (AGI; Model 7541; Ace Glass Inc.), midget impingers (PN: 225-36-1; SKC Inc.), 25 mm gelatin filters (PN: 12602-25-ALK; Sartorius Stedim Plastics GmbH.) in delrin filter holders (PN 1109, Pall Corp.), and a Mercer cascade impactor (PN MCR-3500, In-Tox Products). AGIs were selected for evaluation as they are a common collection device utilized in many published studies with *B*. *pseudomallei* [21–24,26, 27,31–33]. Midget impingers were selected as a liquid sampler with the potential for more gentle collection than the AGI. While the AGI accelerates particles to sonic velocity through a critical orifice, the midget impinger can be operated at lower flow/velocity. Gelatin filters were selected for comparison based on their ease of use and past use in studies with aerosolized *B*. *pseudomallei* [8,22,32]. Additionally, gelatin filters do not contain any glass components, which can be an important safety consideration in the biocontainment laboratories required for work with *B*. *pseudomallei*. The Mercer cascade impactor is a compact stainless-steel collection device that fractionates aerosol particles based on their aerodynamic size, allowing estimation of both the concentration and the particle size distribution of the sampled aerosol.

Midget impingers were loaded with 10 mL PBST and operated at a target airflow of 1 Lpm, regulated with a critical orifice (O’Keefe Controls). AGIs were loaded with 10 mL PBST and operated at 6 Lpm using the critical orifice intrinsic to the sampler. Prior to use, the stainless steel impactor collection discs for the Mercer cascade impactor were coated with a thin layer of polyethylene glycol (PEG; MilliporeSigma) to aid capture of aerosol particles. The impactor was operated at 3.5 Lpm, and material collected on impactor discs during sampling was recovered by placing collection discs into separate tubes containing 5 mL PBST and briefly vortexing. Gelatin filters were operated at a target flow of 1 Lpm, regulated with a critical orifice (O’Keefe Controls). Material collected on gelatin filters was re-suspended by dissolving each filter in 10 mL of PBST at approximately 37°C and vortexing briefly to facilitate dissolution.

### Assessment of physical and biological collection efficiencies

Tests were conducted with fluorescent PSL microspheres to assess the physical performance of the sampling devices and separately with *B*. *pseudomallei* to assess their ability to preserve bacterial viability. All tests with *B*. *pseudomallei* were conducted with the aerosol test system installed in a Class III Biosafety Cabinet. Six replicate tests were conducted with each combination of suspension liquid and analyte. Sampler and system airflows were measured prior to each test using a thermal mass flow meter (PN 4043, TSI, Inc.). For each test, a single sampler of each type was attached to one of the ports on the sampling chamber (Fig 1) and an aerosol containing either *B*. *pseudomallei* or 1 μm fluorescent PSL microspheres in either LB or LB4G was generated with the Collison nebulizer into the test system. Following a ten-second equilibration period after initiation of aerosol generation, sampler airflow was engaged and the samplers collected aerosol for ten minutes.

For each test, the aerosol concentration (C_aero_) of either *B*. *pseudomallei* or the PSL tracer measured at each port was calculated according to Eq 1, where *C*_*s*_ is the analyte concentration in the liquid recovered from the sampler, *V*_*s*_ is the volume recovered from the sampler, *Q*_*s*_ is the sampler air flow rate, and *t*_*s*_ is the sampling duration.

$$C_{aero}=\frac{C_{s}∙V_{s}}{Q_{s}∙t_{s}}$$ Eq 1

For each sampler, the *B*. *pseudomallei* aerosol concentration measured in each test was divided by the mean tracer aerosol concentration from the same sampler type to normalize the culturable *B*. *pseudomallei* aerosol concentration for sampler-specific physical inefficiencies, such as low collection efficiency, re-aerosolization from the sampler, or incomplete recovery of collected material from the sampler. This ratio represents a relative measure of the ability of a given sampler to preserve the viability of collected *B*. *pseudomallei*.

The median aerodynamic diameter and geometric standard deviation (GSD) of the aerosol in each test were calculated using the fluorescence or *B*. *pseudomallei* concentration data from the Mercer cascade impactor stages using a two point interpolation of the cumulative concentration as described previously [42]. Results are reported as activity median aerodynamic diameters (AMAD) rather than mass median aerodynamic diameters (MMAD) as the calculations are based on the activity of the material collected on each stage rather than its mass.

Sample concentrations, aerosol concentrations, and size parameters were calculated using Microsoft Excel 2013. Aerosol concentrations were compared using 2-way ANOVA and Tukey’s post-test with suspension medium and sampler as factors using GraphPad Prism version 6.03 (GraphPad Software). Particle size parameters were compared using 2-way ANOVA with suspension medium and analyte as factors. An alpha level of 0.05 was used as the criterion for statistical significance. All values are presented as mean ± standard deviation.

## Results

**Fig 2:**
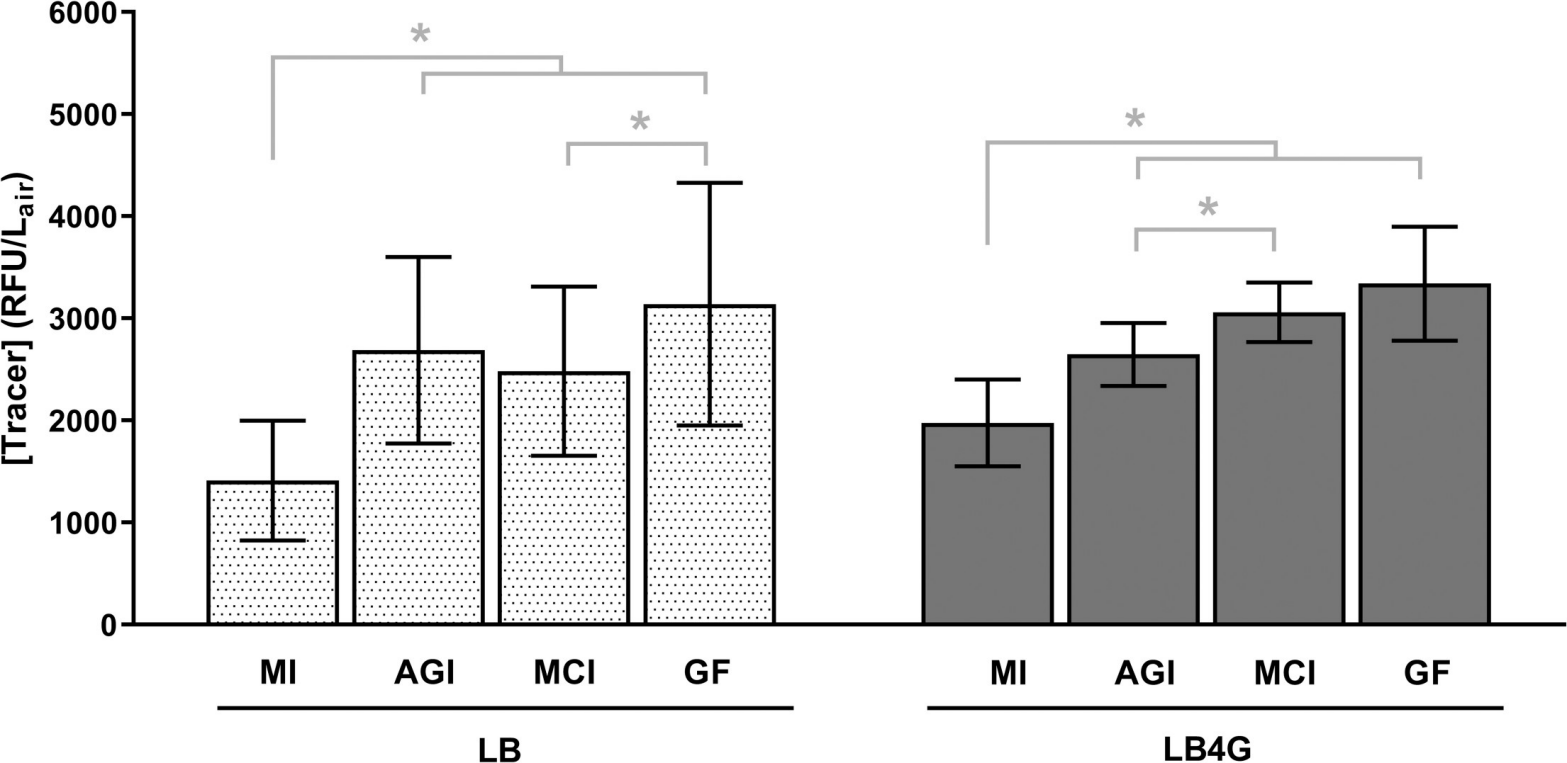
Tracer aerosol concentrations. Aerosol concentrations varied significantly by sampler type (P<0.0001), but neither the suspension medium nor the interaction of sampler and suspension medium were significant factors (P = 0.3977 and P = 0.0961, respectively). Tracer aerosol concentrations measured by midget impingers were lower than all other samplers for aerosols of both LB and LB4G. Small but significant differences were present between the Mercer cascade impactor and either the gelatin filters (LB aerosols), or the AGI (LB4G aerosols). Significant differences by Tukey’s multiple comparisons post-test are indicated by an asterisk. MI = midget impinger, AGI = All Glass Impinger, MCI = Mercer cascade impactor, GF = gelatin filter.

Aerosol concentrations of 1 μm fluorescent microspheres measured with each sampler type are shown in Fig 2. Aerosol concentrations varied significantly by sampler type (P<0.0001), but neither the suspension medium nor the interaction of sampler and suspension medium were significant factors (P = 0.3977 and P = 0.0961, respectively). Concentrations measured by the midget impinger were significantly lower than all other samplers, indicating a lower overall physical collection efficiency for this sampler. The concentrations measured by the AGI, Mercer cascade impactor, and gelatin filter were similar to each other, although small but significant differences were observed between the Mercer cascade impactor and the gelatin filter with LB aerosols (P = 0.0467) and between the AGI and the Mercer cascade impactor with LB4G aerosols (P = 0.0028).

**Fig 3:**
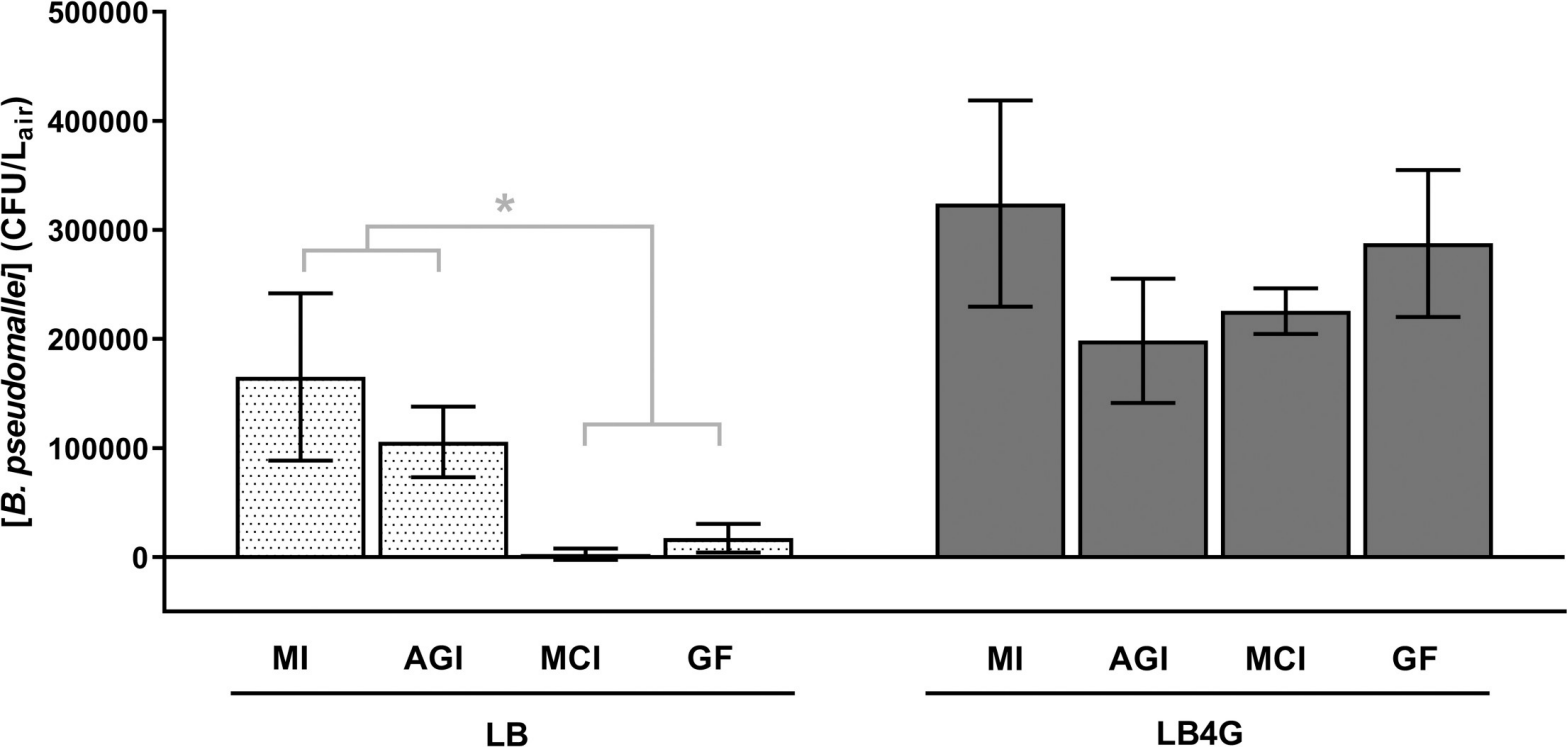
*B*. *pseudomallei* aerosol concentrations. For aerosols generated from *B*. *pseudomallei* suspended in LB, the Mercer cascade impactor and gelatin filter measured significantly lower aerosol concentrations than the AGI and midget impingers. However, for aerosols generated from *B*. *pseudomallei* suspended in LB4G, all four samplers performed equivalently. Significant differences by Tukey’s multiple comparisons post-test are indicated by an asterisk. MI = midget impinger, AGI = All Glass Impinger, MCI = Mercer cascade impactor, GF = gelatin filter.

Aerosol concentrations of *B*. *pseudomallei* measured with each sampler type are shown in Fig 3. Suspension medium, sampler type, and the interaction between parameters were all significant factors when compared using two-way ANOVA (P<0.0025 for all comparisons). *B*. *pseudomallei* aerosol concentrations were significantly higher when aerosolized in LB4G than in LB. No significant differences were observed between the aerosol concentrations measured by any of the samplers for LB4G aerosols. For LB aerosols, there was not a significant difference between aerosol concentrations measured with the AGI and midget impinger. However, the concentrations measured with the AGI and midget impinger were significantly higher than those measured with either the Mercer cascade impactor or the gelatin filter (P<0.0205 for all comparisons).

**Fig 4:**
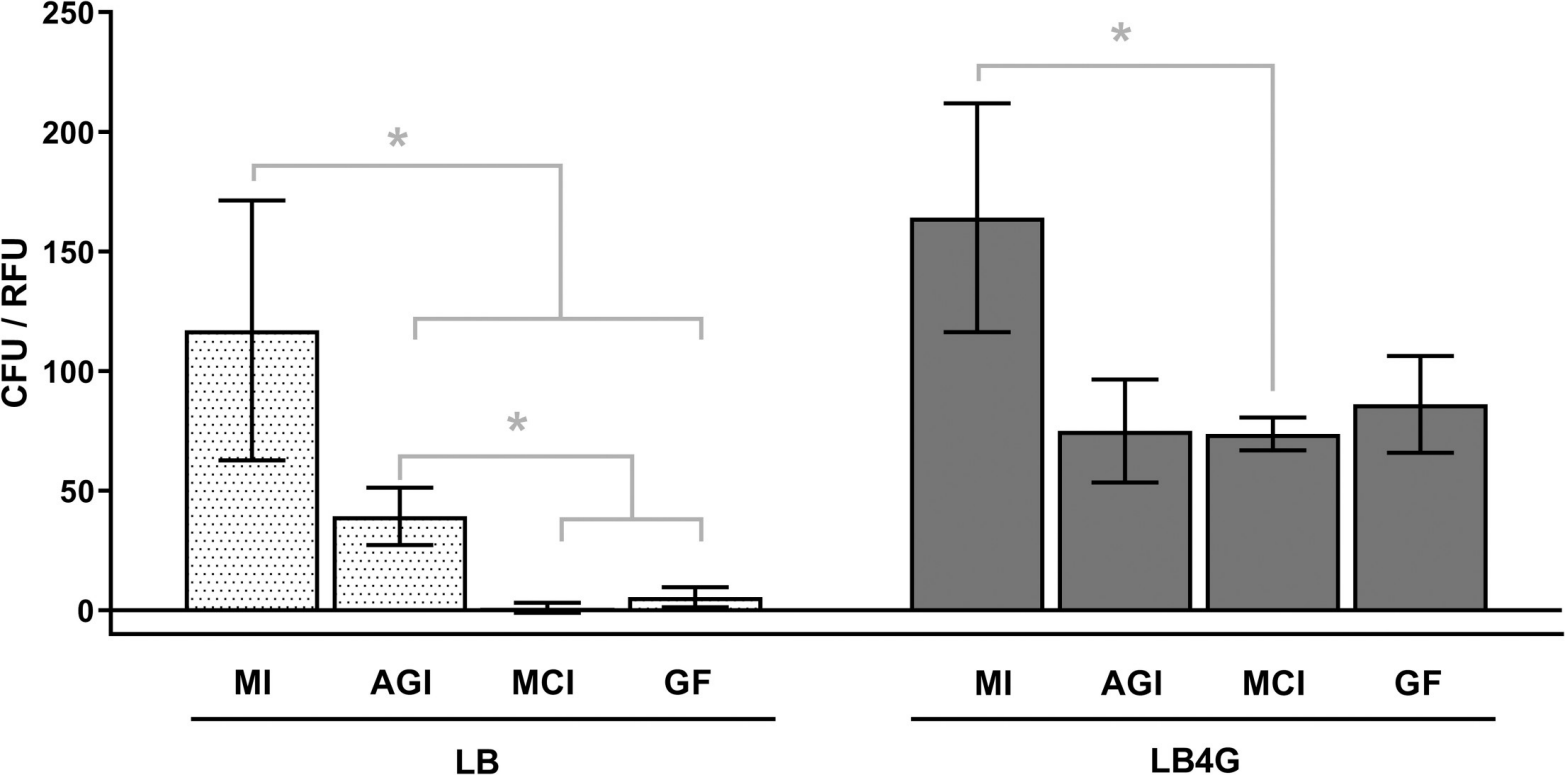
Aerosol Concentration Ratios. The aerosol concentrations of *B*. *pseudomallei* measured with each sampler were normalized for the physical losses by taking the ratio of *B*. *pseudomallei* concentration to tracer aerosol concentration measured for each sampler type. The results of 2-way ANOVA demonstrate that both suspension medium and sampler type, but not their interaction, were significant factors (P<0.0001, P<0.0001, and P = 0.1813, respectively). Significant differences by Tukey’s multiple comparisons post-test are indicated by an asterisk. MI = midget impinger, AGI = All Glass Impinger, MCI = Mercer cascade impactor, GF = gelatin filter.

The ratio of the aerosol concentration of *B*. *pseudomallei* to the aerosol concentrations of 1 μm fluorescent microspheres measured by each sampler was used to normalize for physical losses and provide a relative measure of the ability of a given sampler to preserve the viability of collected *B*. *pseudomallei* (Fig 4). For these ratios, both the suspension medium and sampler type were significant factors (P<0.0001 for both), but the interaction between suspension medium and sampler type was not (P = 0.1813). Ratios for each sampler were higher with aerosols generated from LB4G than LB. For both LB and LB4G aerosols, the largest observed difference in ratios between samplers was between the Mercer cascade impactor and the midget impinger. For LB4G aerosols, this difference was approximately 2-fold, whereas for LB aerosols the difference was over 100-fold.

Particle size distributions estimated using Mercer cascade impactors are shown in Table 1. By 2-way ANOVA, the suspension medium had a small but significant effect on the AMAD (P = 0.0317), but neither the analyte nor the interaction between analyte and suspension medium was a significant factor (P = 0.8450 and 0.5073, respectively). There was not a significant difference between the GSDs as a function of suspension medium, analyte, or their interaction. (P = 0.2557, 0.9941, and 0.2035, respectively).

**Table 1:** Particle size distributions measured by Mercer cascade impactor. The suspension medium had a small but significant effect on the AMAD (P = 0.0317), but neither the analyte nor the interaction between analyte and suspension medium was a significant factor. There was not a significant difference between the GSDs as a function of suspension medium, analyte, or their interaction.

Liquid	Analyte	AMAD (μm)	GSD
LB	1 μm PSL microspheres	1.87 ± 0.37	1.59 ± 0.12
	*B*. *pseudomallei*	1.92 ± 0.32	1.65 ± 0.17
LB4G	1 μm PSL microspheres	1.59 ± 0.22	1.59 ± 0.07
	*B*. *pseudomallei*	1.68 ± 0.03	1.53 ± 0.03

The aerosol concentrations measured by each sampler and the particle size distribution parameters determined from the Mercer cascade impactor data for each test are available in the supplementary information file (S1 Data).

## Discussion

Previous studies have provided evidence that aerosol transmission may contribute to the spread of melioidosis, although the relative contribution of this pathway to overall disease transmission is not known. Accurate measurements of viable airborne *B*. *pseudomallei* concentrations in field and laboratory studies are important for assessing the hazard posed by this transmission pathway. However, such measurements are dependent on the ability of an aerosol sampler to collect and preserve the viability of bacteria, and few studies have examined the performance of aerosol sampling devices with *B*. *pseudomallei* to inform sampler selection or facilitate comparisons between studies. The present study examined the performance of four commonly utilized laboratory aerosol samplers with aerosols of *B*. *pseudomallei* 1026b generated from suspensions in media with and without supplemental glycerol. Results demonstrated that viable bacterial aerosol concentrations were dependent upon both the type of sampler used and the composition of the suspension medium from which the aerosols were generated.

For *B*. *pseudomallei* aerosolized from suspensions in LB broth supplemented with glycerol, the aerosol concentrations of viable bacteria measured with an AGI, midget impinger, gelatin filter, and Mercer cascade impactor were equivalent. This finding is consistent with a previous study which found no differences in the performance of AGIs, gelatin filters, and Mercer cascade impactors for measuring aerosol concentrations of *B*. *pseudomallei* suspended in tryptone broth with 4% glycerol, a formulation similar to the glycerol-supplemented LB broth used in the present study [22]. However, for aerosols generated from suspensions without supplemental glycerol in the present study, measured aerosol concentrations of *B*. *pseudomallei* were dependent on the type of sampler used, with both liquid impingers resulting in significantly higher concentrations than either the Mercer cascade impactor or the gelatin filter.

In addition to tests with *B*. *pseudomallei*, tests were also conducted with 1 μm fluorescent PSL microspheres as a tracer to assess the relative physical performance of the samplers. Differences in tracer aerosol concentrations were observed between the different samplers, but measured concentrations were not affected by the type of suspension medium. This result was expected given the similarity in particle size distributions for aerosols generated from the different media. Comparisons of the ratios of *B*. *pseudomallei* to tracer aerosol concentrations demonstrated that significant differences existed in the ability of the different samplers to preserve the culturablity of collected bacteria, and that these differences were dependent on both sampler type and suspension medium. Notably, while the lower aerosol concentrations of the physical tracer measured by the midget impinger for both media suggest that it is less physically efficient than the other samplers for this particle size distribution, the concentration ratio values suggest that it is better at preserving the viability of collected bacteria than the other samplers. These differences effectively cancelled each other out, a finding that would not have been apparent by only comparing concentrations of viable *B*. *pseudomallei*.

In the present study, liquid impingers were significantly better than the gelatin filter or Mercer cascade impactor at preserving the culturability of collected bacteria aerosolized from LB broth, suggesting that the microorganism may be sensitive to desiccation post-collection within the sampler. This effect was not evident when supplemental glycerol was present in the initial suspension medium, suggesting that the effects of desiccation are dependent on the composition of the aerosol particles. Glycerol is essentially non-volatile at the conditions tested in the present study, and may have protected the organism against desiccation in the gelatin filter and Mercer cascade impactor. These observations are consistent with previous studies that have shown both that bacterial survival during aerosolization is affected by the composition of the initial suspension medium [43–45], and that glycerol can have protective effects on microorganisms in aerosols [46]. While the suspension liquids used in the present study were laboratory media, the observed sensitivity to desiccation is consistent with published reports indicating that natural substrates which facilitate water retention, such as sputum or clay soils, tend to preserve viability of *Burkholderia spp*. [11,47]. Additionally, these results suggest that for field studies of *B*. *pseudomallei* where the composition of aerosol particles is unknown, use of a collection device with a liquid collection medium may provide the best chance of detecting viable bacteria.

*B*. *pseudomallei* is known to enter a viable-but-not-culturable (VBNC) state in response to certain environmental stressors [48]. If the process of aerosolization and/or sampling had induced a VBNC state in the present study, this occurrence would have been indistinguishable from reductions in viability with the culture-based assay that was used. However, while the time required for *B*. *pseudomallei* to transition to a VBNC state has not been well characterized, it has been shown for other bacteria to take hours to days [49]. *B*. *pseudomallei* aerosol tests in the present study were only ten minutes in duration, and the dry samplers were recovered into liquid aliquots within ten minutes of the completion of each test. It is therefore unlikely that transitions to a VBNC state contributed significantly to reductions in aerosol concentrations measured by the samplers in this study, although further testing is needed to confirm this.

Data from the present study demonstrate that the composition of the initial suspension medium can significantly influence comparisons of sampler performance with *B*. *pseudomallei*, and suggest that the results of previous studies may not be applicable to all cases of *B*. *pseudomallei* aerosol collection and analysis. Consideration of such factors is important for studies involving this microorganism, including those attempting to quantify inhaled doses of *B*. *pseudomallei* in animal models of disease. For example, if such a study utilized LB broth as the suspension medium for aerosol generation and a Mercer cascade impactor for sampling, the estimated inhaled dose would be expected to be underestimated by a factor of approximately 100-fold compared to the estimate expected if a liquid impinger was utilized.

Other parameters that were not evaluated in this study also have the potential to impact the performance of sampling devices, including the airflow rate and duration used for sampling, the collection medium used for sampling, the temperature and humidity of the test system, and differences in bacterial growth phase prior to aerosolization [17,50–55]. Furthermore, while relative performance for collecting and preserving bacterial viability is an important consideration when selecting an aerosol sampling device, other factors should be considered as well, including safety, ease-of-use, and durability. For instance, while the liquid impingers in the present study performed better than the gelatin filter and Mercer cascade impactor for aerosols generated from LB suspensions, their glass construction may not be sufficiently durable for some field applications, and may present a safety hazard in biocontainment environments. Additionally, sampling studies for *B*. *pseudomallei* in field or clinical settings may benefit from the use of samplers that operate at higher air-flow rates than the laboratory-scale samplers assessed in the present study, in order to maximize the probability of detecting the organism. Based on the results of the present study, it is likely that there would be a higher probability of culturing *B*. *pseudomallei* from air samples in such studies if the sampling device collected directly into a liquid matrix. However, additional laboratory studies with higher-flow air samplers would be required to confirm this hypothesis.

Results from the present study suggest that any of the four evaluated samplers could be used to measure viable bacterial aerosol concentrations with equivalent accuracy in future studies involving *B*. *pseudomallei* aerosolized from suspensions with supplemental glycerol. However, studies without added glycerol in the aerosolization suspension medium would obtain more accurate measurements of viable *B*. *pseudomallei* aerosol concentrations using a liquid impinger instead of a filter or impactor. Additionally, collection into liquid may provide the best chance to detect viable *B*. *pseudomallei* in field or clinical sampling studies where the organism may be present in aerosol particles of unknown chemical composition.

## Supporting information

S1 Data Raw Data for aerosol concentrations and particle size distributions. (PDF)
