# Supplementary material for: Evaluation of four sampling devices for Burkholderia pseudomallei laboratory aerosol studies
Source: PLoS Negl Trop Dis. 2021 Feb 1;15(2):e0009001. doi: 10.1371/journal.pntd.0009001 (PMC7850477; doi:10.1371/journal.pntd.0009001)
Supplement: S1 Data — (PDF) [file pntd.0009001.s001.pdf]

Test	Analyte	Initial Suspension Liquid	Aerosol Concentrations (RFU/L_{air} or CFU/L_{air}, as applicable)				Particle Size Data	
			Midget impinger	All glass impinger	Mercer cascade impactor	25mm Gelatin filter	AMAD (µm)	GSD
1	1 µm PSL microspheres	LB-Lennox Broth	8.13E+02	1.91E+03	1.78E+03	1.90E+03	1.38	1.46
2	1 µm PSL microspheres	LB-Lennox Broth	1.33E+03	3.09E+03	3.04E+03	3.71E+03	1.82	1.72
3	1 µm PSL microspheres	LB-Lennox Broth	1.77E+03	3.02E+03	3.14E+03	3.94E+03	1.79	1.71
4	1 µm PSL microspheres	LB-Lennox Broth	8.25E+02	1.36E+03	1.32E+03	1.49E+03	NC*	NC*
5	1 µm PSL microspheres	LB-Lennox Broth	1.38E+03	2.82E+03	2.25E+03	3.30E+03	2.41	1.55
6	1 µm PSL microspheres	LB-Lennox Broth	2.35E+03	3.92E+03	3.36E+03	4.50E+03	1.94	1.50
7	1 µm PSL microspheres	LB-Lennox Broth + 4% Glycerol	2.21E+03	2.98E+03	3.36E+03	3.45E+03	1.99	1.69
8	1 µm PSL microspheres	LB-Lennox Broth + 4% Glycerol	1.33E+03	2.17E+03	2.51E+03	3.32E+03	1.42	1.62
9	1 µm PSL microspheres	LB-Lennox Broth + 4% Glycerol	2.09E+03	2.62E+03	3.16E+03	2.38E+03	1.66	1.64
10	1 µm PSL microspheres	LB-Lennox Broth + 4% Glycerol	2.40E+03	2.83E+03	3.11E+03	3.20E+03	1.59	1.57
11	1 µm PSL microspheres	LB-Lennox Broth + 4% Glycerol	2.25E+03	2.87E+03	3.19E+03	3.62E+03	1.44	1.55
12	1 µm PSL microspheres	LB-Lennox Broth + 4% Glycerol	1.58E+03	2.41E+03	3.04E+03	4.07E+03	1.42	1.50
13	*B. pseudomallei* 1026b	LB-Lennox Broth	1.68E+05	1.08E+05	0.00E+00	8.49E+03	NC†	NC†
14	*B. pseudomallei* 1026b	LB-Lennox Broth	1.12E+05	1.02E+05	9.33E+02	3.20E+04	1.92	1.53
15	*B. pseudomallei* 1026b	LB-Lennox Broth	8.38E+04	6.84E+04	6.70E+02	2.12E+04	2.38	1.47
16	*B. pseudomallei* 1026b	LB-Lennox Broth	2.80E+05	1.63E+05	1.31E+04	1.07E+04	1.52	1.88
17	*B. pseudomallei* 1026b	LB-Lennox Broth	1.16E+05	8.40E+04	9.13E+02	1.08E+02	1.75	1.78
18	*B. pseudomallei* 1026b	LB-Lennox Broth	2.32E+05	1.08E+05	3.02E+02	3.15E+04	2.02	1.59
19	*B. pseudomallei* 1026b	LB-Lennox Broth + 4% Glycerol	2.84E+05	2.42E+05	2.47E+05	2.39E+05	1.69	1.49
20	*B. pseudomallei* 1026b	LB-Lennox Broth + 4% Glycerol	5.14E+05	1.18E+05	2.25E+05	2.55E+05	1.70	1.54
21	*B. pseudomallei* 1026b	LB-Lennox Broth + 4% Glycerol	2.55E+05	1.58E+05	1.88E+05	2.50E+05	1.69	1.55
22	*B. pseudomallei* 1026b	LB-Lennox Broth + 4% Glycerol	2.99E+05	2.59E+05	2.21E+05	4.16E+05	1.63	1.53
23	*B. pseudomallei* 1026b	LB-Lennox Broth + 4% Glycerol	3.02E+05	2.41E+05	2.43E+05	2.57E+05	1.68	1.50
24	*B. pseudomallei* 1026b	LB-Lennox Broth + 4% Glycerol	2.92E+05	1.74E+05	2.29E+05	3.10E+05	1.72	1.58

*Mercer cascade impactor stages #5 and 6 were accidentally recovered in the same tube. While this does not affect the determination of the total amount recovered from this sampler, it does affect particle size distribution calculations. Therefore, no size parameters were calculated for this test.

†No culturable bacteria were recovered from the Mercer cascade impactor for this test. Therefore, no size parameters were calculated for this test
